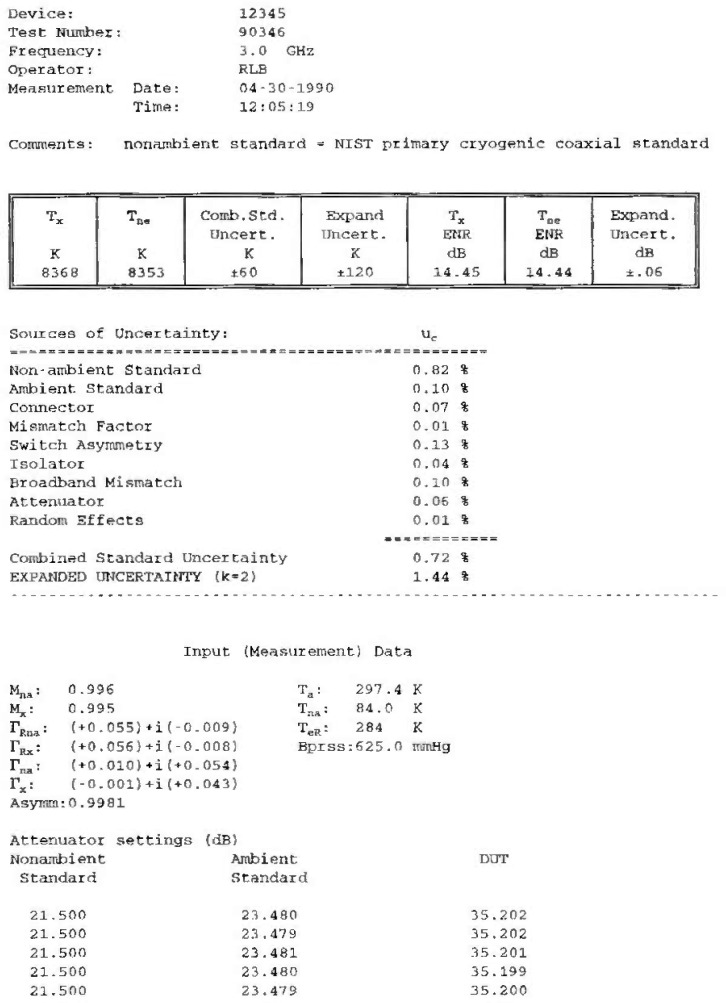# A Null-Balanced Total-Power Radiometer System NCS1

**DOI:** 10.6028/jres.099.005

**Published:** 1994

**Authors:** Sunchana P. Pucic

**Affiliations:** National Institute of Standards and Technology, Boulder, CO 80303-3328

**Keywords:** calibration system, noise standard, noise temperature, null-balanced, radiometer, total power, waveg-uide-below-cutoff attenuator

## Abstract

A recently developed radiometer system NCS1 is used to calibrate thermal noise temperature at any frequency between 1.0 GHz and 12.0 GHz. Any cryogenic noise source can be measured; the upper limit of noise temperatures measured without a loss of accuracy is estimated to be about 10^5^ K. For a typical hot noise source with the noise temperature of 8400 K and a reflection coefficient magnitude of 0.1, the expanded uncertainty is *≈* 1.8%, and the system sensitivity *≈* 2 K. Implemented in Type N connector, it can be easily modified to calibrate noise sources with other coaxial connectors or waveguide flanges.

## 1. Introduction

The Noise Calibration System Model 1 (NCS1), recently built at the National Institute of Standards and Technology, was designed to fill, in the field of thermal noise calibrations, the two overriding needs of calibration laboratories: the highest quality measuring capabilities and a price that is not prohibitive. Currently available commercial systems are not sufficiently sensitive to deliver the adequate accuracy, while the calibration radiometer systems developed previously at NIST may be too expensive to be duplicated by smaller laboratories.

The NCS1 is broadband, accurate, precise, and relatively inexpensive. Its sensitivity is adequate for its intended purpose of calibrating typical hot and cold laboratory and field noise sources (1.9 K with a 8400 K source, 0.09 K with a nominal 77 K cold load). Access to a vector network analyzer is required for reflection coefficient measurements.

The NCS1 can be easily modified to cover wider frequency and dynamic ranges, to accept noise sources with different connectors (or waveguide flanges) than the present Type N configuration, and to have a different null indicator.

## 2. Brief Description

The NCS1 consists of two noise standards and a null-balanced, total-power radiometer. In this implementation, one of the two standards is the NIST primary coaxial standard at the cryogenic temperature of liquid nitrogen; however, a calibrated commercial noise source may be substituted without significantly affecting performance [1]. The other standard is a commercial lossy termination held at ambient temperature.

The NCS1 is equipped with three switch-selectable input ports to accommodate the two standards and an unknown noise source, the device under test (DUT). Switching is controlled from a system control panel by the operator. Since different paths through the switch result in slightly different losses, the switch asymmetry is evaluated (by a separate vector network analyzer), and used to calculate a correction factor (the “asymmetry correction”).

A vector network analyzer is also used to evaluate reflection coefficients of the three NCS1 input ports, the nonambient standard, and the DUT. The results are used to calculate the second of the two correction factors (the “mismatch correction”).

The measured values of the NCS1 input ports and the nonambient standard reflection coefficients are stored in look-up tables used by the NCS1 software and need to be only periodically reevaluated.

The radiometer uses heterodyning to economically cover a broad frequency range, to facilitate frequency selectivity, and to allow for elective future expansion. Since RF sections of the radiometer require bandlimiting to an octave each (Sec. 3.3), there are four front ends corresponding to the four octaves covered (1–2 GHz, 2–4 GHz, 4–8 GHz, and 8–12 GHz). Selection of the appropriate front end is achieved from the system control panel.

A double-balanced mixer downconverts the RF noise to the 30 MHz IF frequency. Both sidebands are used; each has a bandwidth of about 4 MHz. Most of the system gain is achieved in the IF section. A precision waveguide-below-cutoff (WBCO) attenuator attenuates the different noise powers generated by the two standards and the DUT to the same power, thereby achieving a balanced operation mode.

An unbiased square-law diode serves as a detector. Nulling and resolution control are implemented at the post-detection stage. A strip-chart recorder functions as a low-cost null indicator.

**Fig. 1:**
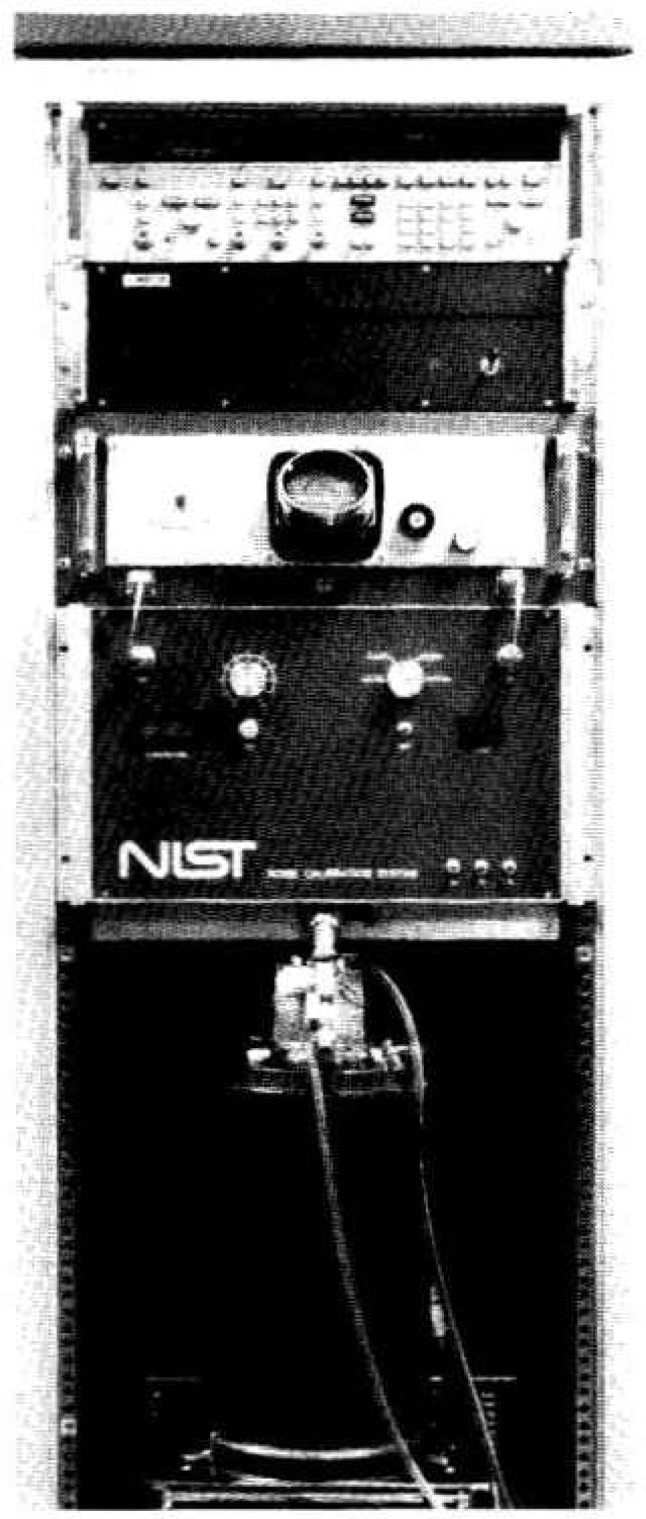
Noise calibration system 1.

Excluding the NIST primary coaxial standard, the NCS1 is constructed of commercially available components and housed in an ordinary equipment rack (Fig. 1). In order to minimize drifting due to ambient temperature variations, the radiometer and the ambient temperature standard are contained in a temperature-controlled enclosure. The temperature control is achieved by a small commercial water circulator housed in the same rack. All components are fastened to and are in good thermal contact with a brass plate, which is held at a uniform temperature by circulating water. Heat-generating components (amplifiers and mixers) have been positioned downstream from passive components. Off-line computer support is written in BASIC.

## 3. Noise Calibration System Model 1

**Fig. 2:**
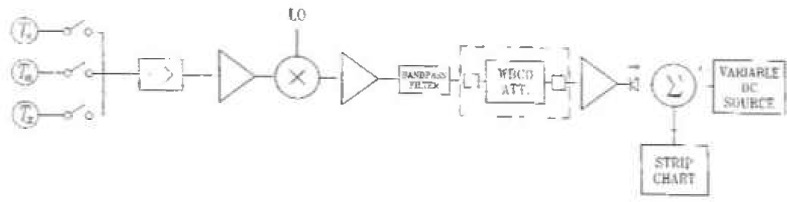
NCS1 block diagram.

A block diagram of the Noise Calibration System Model 1 is shown in Fig. 2. In the following sections, the two standards are briefly overviewed, and the radiometer is described in more detail.

### 3.1 Cryogenic Temperature Standard

The NIST primary coaxial cryogenic noise standard is described in [2]. Briefly, it consists of a lossy termination with its connecting network (a beaded air-line, an adaptor, and the connector), a water jacket surrounding the output section, and a Dewar holding the liquid nitrogen at atmospheric pressure. All components are housed in an electrically conductive enclosure.

The temperature of the termination is uniform and held constant at the temperature of the liquid nitrogen, which is corrected for the small variations in the static pressure due to the head loss to evaporation, and the barometric pressure. The water circulating within a water jacket locks the temperature of the output section of the connecting network (the adaptor and the connector) to the ambient temperature. The temperature gradient along the length of the coaxial air-line is assumed to be linear with distance between the cryogenic termination and the room temperature connector.

The noise temperature at the output of the primary cryogenic standard, *T*_s_, has therefore three calculable components: the major contribution of the lossy termination at the cryogenic temperature, and the minor contributions from the temperature transitional region (the low-loss coaxial air-line), and from the ambient temperature region (the low-loss adapter and connector). The expression used to calculate the output temperature [2] is incorporated into the NCS1 software.

### 3.2 Ambient Temperature Standard

The ambient temperature standard is a lossy termination whose temperature is held constant by circulating water. Its temperature is measured by a calibrated thermometer. Since every component of this standard is at the same (ambient) temperature, the losses in the connecting network are irrelevant. The output noise temperature of the ambient standard is simply its thermodynamic temperature *T*_a_.

### 3.3 NCS1 Radiometer

**Fig. 3:**
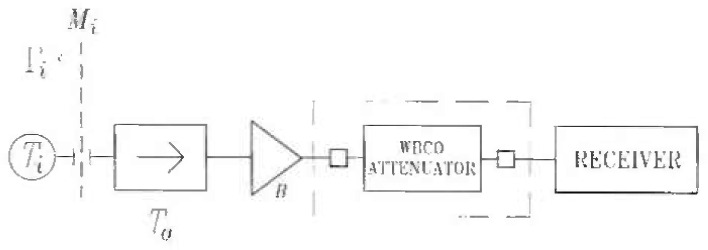
Simplified system block diagram.

The principle of the NCS1 is illustrated by the simplified block diagram in Fig. 3. The simplified radiometer consists of only four components: an isolator, a RF amplifier, a WBCO attenuator, and a receiver containing a square law detector. The input switch is omitted, and the noise sources attach directly to the single input port.

The radiometer input signal is the broadband noise generated by the three noise sources: the two noise standards and the DUT, Each of the three sources is sequentially attached to the radiometer input port, and the noise power is adjusted by the attenuator so that the receiver balances in all three cases.

Following [3] and [4], the available noise temperature *T*_x_ of the DUT can be calculated by comparing it to the available noise temperatures *T*_s_ and *T*_a_ of the two standards, according to

$$T_{x}=T_{a}+\frac{M_{s}}{M_{x}}(T_{s}-T_{a})\frac{10^{-(A_{a}-A_{x})/10}-1}{10^{-(A_{x}-A_{s})/10}-1},$$ (1)

where *M*_s_*/M*_x_ is the mismatch correction at the input reference plane, and *A*_a_, *A*_s_ and *A*_x_ are the attenuator settings (in dB) needed for balance.

Three features help convert the idealized radiometer above into a workable instrument:

- In order to protect the connectors and facilitate the operation, an electrically operated single-pole, triple-throw coaxial switch, the input switch, is placed at the system input, effectively creating three separate, dedicated system input ports. The nonambient and ambient temperature standards and the DUT are then sequentially *switched into* the radiometer.
  There are two consequences of that arrangement: the different paths through the input switch result in different losses and require an *asymmetry* correction, and the finite isolation between the paths cause leakage, which, however, becomes negligible with a proper switch selection.
- In order to economically broaden the frequency coverage, heterodyning is employed. Since the input signal is wideband, it must be bandlimited ahead of the mixer, to avoid intermixing the out-of-band signals. The use of a double-balanced mixer, which suppresses second-order intermixing products, allows the RF sections preceding it to be a full octave wide. The bandlimiting is achieved by means of the RF amplifiers preceding the mixer, since passive filters resonate in their “far out-of-band” region and, therefore, fail to effectively achieve the bandlimiting goal. The same bandlimiting also eliminates problems of the noise signal mixing with harmonically related and out-of-band spurious signals originating in the local oscillator. The input noise signal is downconverted to 30 MHz, the value dictated by the commercial WBCO attenuator. Both sidebands are used, so the measured noise signal power is the *mean* of the powers contained in the two symmetrical bands, 60 MHz apart and filtered to be 4 MHz wide, centered around the LO frequency. The spectrum of the noise sources is assumed to be essentially flat within the resulting 64 MHz averaging interval.
  The fact that the noise ts measured at LO±IF and *not* precisely at the LO frequency, combined with the non-negligible electrical length of the input section in front of the isolator, results in a *broadband mismatch error* [1], [5].
- In order to implement a fully null-balanced system, the post- detection stage of the receiver includes DC nulling circuitry built around an operational amplifier. A strip-chart recorder serves as a null indicator.

**Fig. 4:**
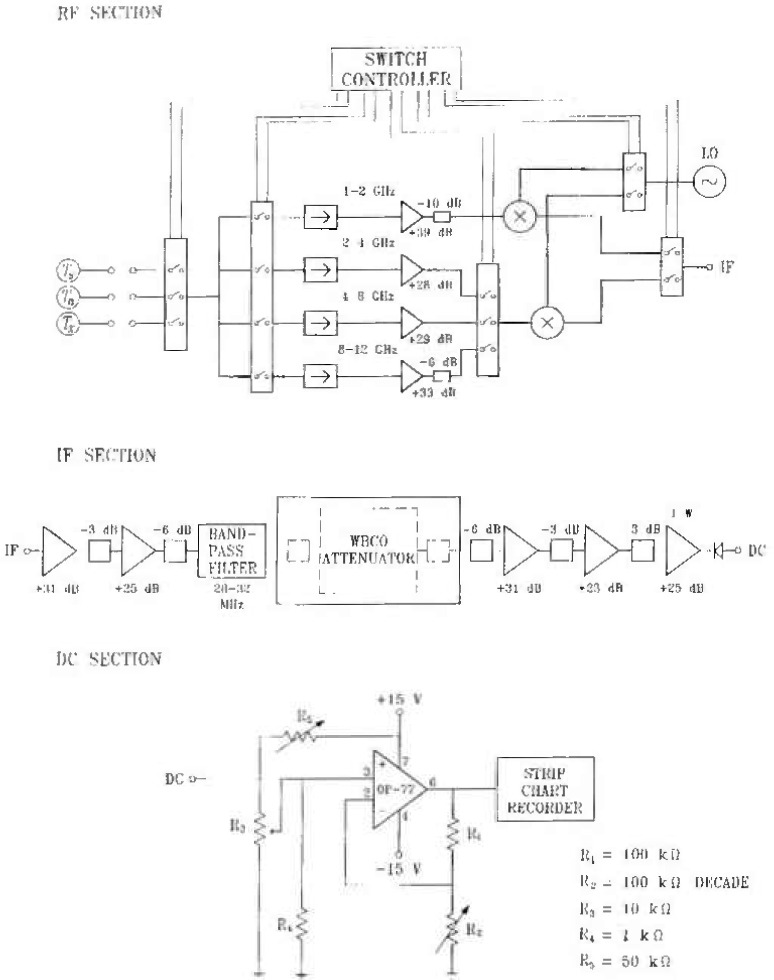
Schematic diagram of NCS1.

Figure 4 shows a schematic diagram of the implemented radiometer.

The radiometer front ends are configured with the help of four switches, mounted on the control panel and labeled by the octaves they form, “1–2,” “2–4,” “4–8,” and “8–12.” The four switches control electrically operated internal switches, which are transparent to the user. There are two mixers in the radiometer, because no mixer covering the full 1 GHz to 12 GHz band was available. The resistive pads in front ends help equalize the signal at the mixer input (since RF amplifiers have all different gains). Numerous 3 dB and 6 dB pads inserted in the IF system improve amplifier stability by controling the internal reflections.

The total IF gain of the system is partitioned between the pre-attenuator and post-attenuator sections. Although any amplifier saturation in the post-attenuator section is inconsequential (so one is tempted to put all the amplifiers there), a certain amount of gain must precede the attenuator for two reasons: to make the noise contribution of the attenuator negligible in comparison with a much stronger (amplified) signal at the attenuator input, and to reduce the attenuator vulnerability to the electromagnetic interference.

#### 3.3.1

The *available* noise temperature *T*_x_ of the DUT is calculated from the system equation [3]

$$T_{x}=T_{a}+\frac{M_{s}}{M_{x}}\cdot \frac{{\eta \mathrm{sw}}_{a}}{{\eta \mathrm{sw}}_{a}}\left(T_{s}-T_{a}\right)\frac{10^{-(A_{s}-A_{r})/10}-1}{10^{-(A_{a}-A_{s})/10}-1},$$ (2)

where, as before, *T*_s_ and *T*_a_. are the available noise temperatures of the two standards, *M*_s_/*M*_x_, is the mismatch correction at the input reference plane, and *A*_s_, *A*_a_, and *A*_x_ are the attenuator settings needed for balance. Furthermore, ${\eta \mathrm{sw}}_{a}$ ${\eta \mathrm{sw}}_{a}$, is the input path asymmetry correction, a consequence of slightly different efficiencies of the two sides of the input switch. Both the mismatch correction and the asymmetry correction are evaluated using a vector network analyzer.

The analysis of uncertainty is presented in detail in [1]. Briefly, there are eight significant standard uncertainty components arising from systematic effects: two pertain to the standards and the rest to the radiometer and the DUT. The contribution of the random effects is small. The combined standard uncertainty is calculated by a root sum of squares (RSS) method. The expanded uncertainty is calculated by multiplying the combined standard uncertainty by the coverage factor of 2. For a typical DUT with a noise temperature of 8400 K and a reflection coefficient of 0.1, the expanded uncertainty is approximately 150 K.

#### 3.3.2 System Sensitivity

The sensitivity (the minimum detectable signal) of a radiometer with negligible short-term gain fluctuations is defined [6,7] as the minimum input signal noise temperature difference required to produce an output signal noise temperature having a signal-to-noise ratio of 1. It is given by

$$\Delta T_{\min}=\frac{T_{\text{in}}+T_{\mathrm{cRD}}}{\sqrt{B\cdot \tau }}$$ (3)

where *T*_in_ is the available noise temperature of the noise source attached to the radiometer input, *t*_crd_ is the effective input noise temperature of the radiometer, *B* is the limiting pre-detection system bandwidth, and *τ* is the integration time of the post-detection circuitry.

The effective input noise temperature of the radiometer has been experimentally shown to vary between 300 K and 800 K. and is higher in the higher frequency bands. The limiting pre-detection system bandwidth *B* is set to 4 MHz by an 8-pole band-pass filter preceding the WBCO attenuator. The integration time of the post-detect ion circuitry *τ* is about 5 s. (The limiting post-detection frequency response, that of the strip-chart recorder, is ≈ 15 Hz, but the trace is visually integrated by the operator over 5 s or so.)

A typical system sensitivity, expressed in kelvins, for a DUT with noise temperature of 8400 K measured at 2 GHz, with *T*_cRD_ of ≈ 350 K, is 1.9 K. Under the same conditions, the system sensitivity for a cold load of (nominally) 77 K is 0.09 K.

#### 3.3.3. Dynamic Range

The dynamic range of the NCS1 is primarily determined by the WBCO attenuator. Based on the calibration data, the attenuator transfer function does not significantly depart from linearity between 20 dB and the highest calibrated value of 75 dB, for a dynamic range of at least 55 dB. However, the NCS1 dynamic range may be limited by active elements preceding the attenuator if driven into saturation, or the leakage of the signal. The verified dynamic range for the implemented radiometer is 15 dB. Additional tests would be needed to determine its full dynamic range.

#### 3.3.4 Operation

##### External equipment needed

A microwave generator having a stable, unmodulated output signal at +10 dBm is required to serve as a local oscillator. Liquid nitrogen and a barometer are needed for the operation of the NIST primary cryogenic standard. Access to a vector network analyzer is also necessary to evaluate the mismatches and asymmetries at the NCS1 input.

##### Front end characterization

Once measured, data on the reflection coefficients and the input switch efficiencies are stored in appropriate computer files and require only periodic checking. The frequency of the periodic checks depends on the calibration load, the previous wear of the input switch, and the extent of mechanical disturbances and ambient temperature variations incurred by the system since the previous check. A change in the real and imaginary parts of the reflection coefficients of the radiometer of ≈ 0.007 and a change in efficiencies of 0.1% require a new front end characterization. The software data update procedure is menu-driven.

##### Preparations for noise measurement

The reflection coefficient of the DUT must be measured before each calibration at all frequencies of interest. The NCS1 hardware, including the water circulator, should be turned on several hours ahead of the measurements in order for the system to reach thermal equilibrium. If the cryogenic standard is the nonambient standard, it must be filled with liquid nitrogen to the appropriate (marked) level. To prevent a possible damage due to freezing, water surrounding its output section must circulate at all times the standard is filled with liquid nitrogen, Manufacturer instructions guide a warm-up period for a DUT and a commercial nonambient standard, if such is used. After selecting the front end corresponding to the measurement frequency, the noise source with the lowest noise temperature (the cryogenic standard, if used with a hot DUT; the ambient standard if the secondary hot standard is substituted for the cryogenic standard) is switched into the input port and the WBCO attenuator adjusted to center the strip-chart trace. The input to the strip-chart recorder is momentarily grounded; no deflection of the center line verifies a true zero input signal.

Because of linearity, the lowest attenuator setting must not be below 20 dB.

The system resolution is checked as follows: the DUT is switched into the radiometer, and the trace brought back to the center by the appropriate attenuator adjustment. A stable baseline is established. The attenuator setting is changed by a small amount *ΔA*, typically 0.01 dB, causing the trace to deflect by a small amount *Δd.* The resolution can be adjusted by varying the gain of the post-detection operational amplifier (using a small decade counter on the front panel labeled “GAIN”).

##### Measurement procedure

A full noise calibration consists of at least five measurement sets. Each set involves switching the nonambient standard, the ambient standard, and the DUT into the system input, and adjusting the WBCO attenuator to maintain the trace on the strip chart centered. The software prompts the operator to input the attenuator dial setting. The resulting DUT noise temperature and associated statistical data are displayed on the monitor, allowing the operator to increase the number of measurements in a set if the standard deviation is unsatisfactorily large (greater than 25 K or so for a 9000 K device).

The system stability and general performance are checked by comparing the nonambient standard traces of each consecutive measurement set. A deflection from the previous position on the strip-chart indicates excessive drift (and should prompt a water circulator check), or some malfunction. The operator can direct the software to disregard the previous measurement set or to keep the set and increase the number of measurements.

The computer printout consists of a single sheet, showing the input data, the calculated noise temperature of the DUT, and the combined standard uncertainty and its components, as well as the expanded uncertainty (Table 1).

## 4. Discussion

The NCS1 has been designed to be an inexpensive system compatible with requirements of laboratories needing the highest achievable accuracy in thermal noise calibrations in the microwave part of the spectrum.

The radiometer is a total-power radiometer, as opposed to a switching (Dicke) radiometer [8,9]. The driving force behind the switching concept is drift. In the laboratory environment, because of the availability of inexpensive water cooling, the NCSl’s sensitivity (≈ 3 K), and the short time necessary to complete a measurement (minutes), drift is not a problem.

Similar to the switching, but unlike the other total-power radiometers at NIST [4], the NCS1 radiometer is a null-balanced instrument. Balancing is achieved in the IF section by a WBCO attenuator. Nulling occurs in the dc section by the operational amplifier circuit (Fig. 4).

Balancing allows the circuitry following the balancing device to be nonlinear, a major advantage. Tight linearity requirements are imposed on a balancing device, though. Based on a theoretical analysis and confirmed by calibration [10], the precision WBCO attenuator used in the system has a remarkably linear functional relationship between the coil separation and output power in a range of at least 55 dB.

The advantage of nulling is not only that the circuitry following the nulling device need not be linear, but also that its frequency response and thermal loading are immaterial [11]. Nulling speeds up the operations, since the strip-chart recorder that follows the nulling circuit in the NCS1 does not require settling time.

The commercial precision WBCO attenuator used in NCS1 is tuned to 30 MHz. The tuning circuit does not, however, provide sufficiently narrow and constant IF bandwidth, so a separate bandpass filter is required. This 4 MHz filter is the band-limiting element in the NCS1.

In practical terms, the commercial availability of a 30 MHz WBCO attenuator determines the IF frequency of the system. Since the uncertainty arising from the broadband mismatch increases rapidly with increasing IF frequency [1], a WBCO attenuator with a lower cutoff frequency is preferable.

A 30 MHz IF frequency also limits the expansion of the NCS1 frequency coverage of bands below approximately 250 MHz, since the frequency selectivity of the measurements would be compromised by the relatively wide 65 MHz averaging interval.

To expand the coverage to frequency bands above 12 GHz, a (secondary) noise standard must replace the NIST coaxial cryogenic standard, which cannot be used above that limit. New RF sections, each no more than an octave wide, would have to be integrated into the system. Frequency-sensitive uncertainties would increase, but there are no theoretical reasons limiting the upward frequency expansion. An alternative coaxial connector or a waveguide flange may be installed to replace the present precision Type N female connector. If the input switch is equipped with additional poles with appropriate connectors, the NCS1 can provide a calibration service in multiple connectors. Each path would need to have its efficiency determined. Care would have to be taken regarding the mechanical and thermal requirements of the input section; because of the broadband mismatch uncertainty the input section of the radiometer must be short, and at the ambient temperature.

The radiometer sensitivity could easily be improved by replacing the present RF amplifiers (noise figure 2.5 dB–1.5 dB, depending on the band), with low-noise amplifiers.

Because of nulling, the requirements on the output device of the NCS1 are low: stability over several minutes required for a measurement, and sufficient sensitivity and resolution. The strip-chart recorder can be replaced by a voltmeter or an A/D converter for on-line processing.

## Figures and Tables

**Table 1:** A calibration report